# Assessment of the Zulfiqar Frailty Scale (ZFS) in Primary Healthcare

**DOI:** 10.3390/jcm13123481

**Published:** 2024-06-14

**Authors:** Abrar-Ahmad Zulfiqar, Emmanuel Andres

**Affiliations:** Internal Medicine Department, Hôpitaux Universitaires de Strasbourg, 67000 Strasbourg, France; emmanuel.andres@strasbourg.fr

**Keywords:** Zulfiqar Frailty Scale, modified SEGA scale, general medicine, elderly subjects, frailty syndrome

## Abstract

Introduction: The primary aim of the study was to validate the Zulfiqar Frailty Scale (ZFS) and examine its concordance with the modified Short Emergency Geriatric Assessment (mSEGA) scale, Part A. Methods: A prospective observational study was conducted in Guadeloupe (France) over a two-month duration (from 20 February to 20 April 2024), involving elderly individuals aged 65 and older, deemed self-sufficient with an ADL (Activities of Daily Living) score exceeding four out of six. Results: Within this community cohort of 98 individuals, averaging 75 years in age, frailty according to the modified SEGA criteria was prevalent in 29%. Frailty according to the “ZFS” score was prevalent in 40%. Key predictors of frailty identified in our study included age, comorbidity (Charlson score), polypharmacy (total number of medications and therapeutic classes), and functional ability (ADL scores). Notably, experiences of falls and hospitalizations within the past six months significantly influenced the classification of frailty according to both ZFS and SEGA scales. Significant associations with the presence of home care aides (*p* < 0.0001), monopodal support test results (*p* < 0.0001), memory impairments (*p* < 0.0001), and recent hospitalizations (*p* = 0.0054) underscored the multidimensional impact of frailty. The Pearson correlation coefficient and its 95% confidence interval between the SEGA and Zulfiqar Frailty Scales stood at 0.73 [0.61: 0.81]. The discernment threshold for frailty was set at three out of six criteria, showcasing a sensitivity of 64% and a negative predictive value of 80%. The area under the curve (AUC) for the Zulfiqar Frailty Scale was reported as 0.8. Conclusion: The “ZFS” tool allows for the detection of frailty with a highly satisfactory sensitivity and negative predictive value.

## 1. Introduction

In their capacity for prevention and screening, the general practitioner holds a central position in recognizing hazardous lifestyle patterns. However, assessing frailty in primary care proves daunting due to the array of definitions and diagnostic instruments at hand. Moreover, their applicability is not consistently tailored for private practice settings. With the goal of unifying professional protocols and facilitating frailty evaluation during general medical visits, we have introduced a screening tool for frailty known as the Zulfiqar Frailty Scale. This scale consists of six elements, outlined in detail in Table 1.

This instrument consists of six components, which have been identified in the literature as significantly and independently correlated with adverse outcomes concerning morbidity and mortality, thus aligning with the definition of frailty markers [2,3]. Nutritional status, Balance and falls, Cognitive function, Social capabilities, Polypharmacy.

The choice of these components was guided by their swift completion and straightforward nature, thus circumventing the necessity for prior training. The inaugural investigation validating our frailty scale was conducted in a general medical practice in Brittany and was published in the *Medicines* MDPI journal [1].

The current gold standard for frailty screening in elderly patients is the Fried Phenotypic Frailty Scale [4], recommended as the initial approach by the American Geriatric Society and the Haute Autorité de Santé (French Health Authority) [5]. However, the practical application of this screening tool in general medicine faces challenges due to the requirement of a dynamometer. Moreover, this scale primarily focuses on frailty within the context of sarcopenia, overlooking psychosocial components.

Canadian teams led by Kenneth Rockwood have conceptualized a Frailty Index [6,7]. Developed based on a multidimensional clinical model of frailty, it encompasses 70 dichotomous items, considering physical factors (e.g., incontinence and cardiovascular conditions), psychological factors, cognitive factors, and the impact on activities of daily living (autonomy). This scale categorizes elderly subjects into seven categories, ranging from “very robust” to “severely frail,” including “well” or “well with treated conditions.” It views frailty as a cumulative variable where each deficit worsens frailty, contrasting with the Fried Scale. Nonetheless, it is not well suited for ambulatory care due to the time constraints of administering 70 items.

The Gerontopôle de Toulouse Frailty Screening Tool (GFST) consists of six closed-ended questions focusing on isolation, weight loss, asthenia, gait disorders, and memory. Additionally, a subjective question is directed at the general practitioner: “Does your patient seem frail to you?” [8,9,10]. The main limitation of this tool is the subjectivity of its items. However, it does not aim to be a scale but rather a screening tool to detect patients needing daily hospital care.

For our study, we utilized the Short Emergency Geriatric Assessment (SEGA) scale, initially developed by a Belgian research team led by Dr. Schoevaerdts, for quick identification of the geriatric profile of elderly patients admitted to the emergency room [11]. The SEGA tool is founded on functional decline risk factors documented in the literature, expert opinions, and data from the DecLIC cohort study (DecLine Investigation Cohort, which examined functional decline factors among 600 patients aged over 70) [11,12]. Thirteen factors were selected and grouped into SEGA Part A, weighted empirically on three levels (0, 1, and 2). It assesses frailty using a 13-item scale, encompassing: medications/mood/perception of health/falls in the previous 6 months/nutrition/associated diseases/mobility/continence/cognitive function/age/place of living/IADL/meals. Each item is graded as either 0 (most favorable state) or 1 or 2 (least favorable state), enabling classification of subjects into three categories: not very frail (score ≤ 8), frail (8 < score ≤ 11), and very frail (score > 11). Part B groups categories of information facilitating or complicating hospital discharge planning. It was administered in the ER by an experienced physician or nurse, taking a relatively short time (under ten minutes), focusing on the patient’s situation fifteen days before admission. The scale is not a geriatric assessment or prognostic tool, yet it correlates with certain geriatric frailty indicators (increased length of stay, geriatric admission, readmission within six months, and mortality rate), thus becoming a risk-based geriatric profile. It was later adopted by the RéGéCa Geriatric Network and the Reims University Hospital, adapted for outpatient use. Dr. Drame’s team at University Hospital of Reims validated Part A on a cohort of 167 home-living elderly patients in 2014 [12].

We are adopting an innovative perspective by combining two complementary frailty assessment tools for elderly individuals. This pioneering approach not only allows us to validate and compare the effectiveness of two distinct methods in various clinical contexts but also to develop more precise and personalized intervention strategies. By integrating the specific features of the ZFS, which offers a multidimensional and rapid evaluation of frailty, with the robustness of the mSEGA, particularly suited for emergency environments, our study aims to enhance the overall management of geriatric patients. Through this approach, we hope to contribute to better clinical outcome prediction, reduce avoidable hospitalizations, and improve the quality of life for elderly individuals. This study is innovative, as it explores new synergies between two frailty assessment tools, paving the way for more integrative and effective clinical practices.

Primary objective: The primary aim of the study was to validate the Zulfiqar Frailty Scale (ZFS) and examine its concordance with the modified Short Emergency Geriatric Assessment (mSEGA) scale, Part A, which is scored out of 26 and consists of 13 elements.

## 2. Patients and Methods

### Methods

To address our research inquiry, a preliminary prospective study was conducted within a General Medicine practice in Guadeloupe over a total duration of 2 months (from 20 February to 20 April 2024).

- Inclusion criteria: Eligible patients were required to be 65 years of age or older, seeking consultation in general medicine, and possessing an Activity of Daily Living (ADL) score of 4 or higher. Patients under 65 years of age and those with an ADL score below 4 were excluded from the study. Patients living in nursing homes were also excluded, as were patients unable to communicate or provide consent.
- Data collection and analysis: The data essential for the study were collected by the General Practitioner during routine consultations. For each patient, both the mSEGA Part A frailty scale and the Zulfiqar Frailty Scale were administered. The information was then anonymized before being submitted for study data compilation.
- Statistical analysis:

Software and Data Presentation

Statistical analyses were performed using R version 4.3.0 through the RStudio interface version 2023.06.1. The choice of this software is grounded in its robust statistical capabilities and wide acceptance in the research community. R provides a comprehensive suite of statistical and graphical techniques, including linear and nonlinear modeling, classical statistical tests, time-series analysis, classification, clustering, and more.

Data Expression

Qualitative variables were expressed in terms of frequencies and percentages per response modalities. This categorical data summarization provides an intuitive understanding of the distribution of different responses. For instance, if evaluating the presence of frailty symptoms, the number and percentage of subjects exhibiting each symptom are presented.

Quantitative variables were presented as means and standard deviations. The mean provides a measure of central tendency, while the standard deviation indicates the variability or dispersion from the mean. This dual presentation ensures a complete picture of the data distribution, essential for understanding the overall trends and deviations within the study sample.

Correlation Analysis

A Pearson correlation matrix was generated to explore correlations between items and total scores of the two scales (Zulfiqar Frailty Scale and mSEGA Part A). Pearson’s r measures the strength and direction of the linear relationship between two variables, ranging from −1 (perfect negative correlation) to +1 (perfect positive correlation). This matrix helps in identifying which items are strongly related and contributes to understanding the coherence and consistency of the scales.

Comparison of Frailty Scales

To evaluate the properties of the Zulfiqar scale, a direct comparison was made with the mSEGA Part A scale, where patients were deemed frail if their score exceeded 8 out of 26. This threshold is based on established clinical cut-offs, ensuring the comparability and relevance of the findings.

ROC Curve and Diagnostic Accuracy

A receiver operating characteristic (ROC) curve was plotted to assess the diagnostic ability of the Zulfiqar Frailty Scale. The area under the curve (AUC) was computed to quantify the overall ability of the test to discriminate between frail and non-frail individuals. An AUC of 0.8 indicates good diagnostic ability, with values closer to 1.0 representing excellent discrimination.

Sensitivity and Specificity

Sensitivity (true positive rate) and specificity (true negative rate) were calculated to determine the accuracy of the scale in identifying frail individuals. Sensitivity measures the proportion of actual positives correctly identified by the test, while specificity measures the proportion of actual negatives correctly identified. These metrics are crucial for evaluating the performance of the frailty scale in different clinical settings.

Predictive Values and Youden Index

The positive predictive value (PPV) and negative predictive value (NPV) were calculated to assess the test’s effectiveness in predicting frailty. PPV is the probability that subjects with a positive screening test truly have the condition, while NPV is the probability that subjects with a negative screening test truly do not have the condition.

The Youden index, calculated as sensitivity + specificity − 1, was used to determine the optimal cut-off point for the frailty scale. This index maximizes the test’s overall ability to correctly classify individuals as frail or non-frail, providing a single statistic that reflects the test’s diagnostic effectiveness.

Significance Level

A significance level of 95% was selected, meaning that for a result to be considered significant, the *p*-value had to be below 0.05. This conventional threshold ensures that the findings are statistically robust, reducing the likelihood of Type I errors (false positives).

By utilizing these comprehensive statistical methods, the study aims to rigorously evaluate the performance and reliability of the Zulfiqar Frailty Scale, contributing valuable insights to the field of geriatric assessment and care.

- Administrative considerations: Informed consent was obtained from all patients included in the study. From a regulatory perspective, the study was registered with the CNIL (National Commission on Informatics and Liberties) with reference number 2227749. The experiment was approved by the Ethics Committee of Ile de France VI and is registered under reference number 2022-A03779-22.

## 3. Results

Population description: throughout the data collection phase, 98 patients aged 65 and above were enrolled, with no instances of refusal recorded.

Justification for the Sample Size of 98

Considering the complex nature of geriatric populations and the multifaceted assessment of frailty, a sample size of 98 provides a more reliable and generalizable dataset due to:

1. Enhanced reliability: a larger sample size reduces the margin of error and increases the precision of correlation estimates.
2. Generalizability: with 98 participants, the study results are more likely to be applicable to a broader population.
3. Subgroup analyses: allows for more detailed analyses across different subgroups (e.g., age ranges and comorbidities).
4. Statistical power: ensures sufficient power to detect meaningful differences and correlations, even in the presence of variability.

The characteristics of the enrolled population are elaborated in Table 2.

### 3.1. Correlation between SEGA and Zulfiqar Frailty Scales

The Pearson correlation coefficient (Pearson’s r) and its 95% confidence interval were reported as 0.73 [0.61: 0.81]. Table 3 and Table 4 display the Pearson correlation matrix, illustrating the correlations among the items of the SEGA and ZFS scales, in addition to geriatric, anthropometric, and iatrogenic criteria.

- Age: The *p*-value (0.0000022) indicated a highly significant positive correlation between age and ZFS score. Older age was significantly associated with higher ZFS scores, implying that older individuals were more likely to be classified as frail according to the ZFS.
- Charlson score: The *p*-value (0.00000405) showed a significant positive correlation between the Charlson comorbidity index and the ZFS score, suggesting that individuals with more comorbid conditions had higher ZFS scores.
- ADL/6: The *p*-value (3.91 × 10^−8^) indicated a significant negative correlation between ADL scores and ZFS scores. Higher ADL scores (indicating better daily living abilities) were associated with lower ZFS scores, suggesting that better functional ability was linked to lower frailty.
- Current weight in kilograms, weight in cm, BMI, and weight 6 months ago in kilograms: The non-significant *p*-values indicated no significant correlation between these variables and the ZFS scores.
- Total number of medications: The *p*-value (8.09 × 10^−9^) showed a significant positive correlation, suggesting that a higher number of medications was associated with higher ZFS scores.
- Total number of therapeutic classes: The *p*-value (3.21 × 10^−8^) indicated a significant positive correlation, similar to the total number of medications, linking polypharmacy to higher frailty scores.
- mSEGA scale grid A (/26): The *p*-value (3.01 × 10^−17^) indicated a very strong positive correlation between the mSEGA and ZFS scores, demonstrating that higher mSEGA scores were significantly associated with higher ZFS scores.

**Table 4:** Pearson correlation coefficients with the SEGA score variable.

Variables	The Pearson Correlation Coefficient	CI	*p*-Value
Age	0.56	[0.4092: 0.6841]	1.74 × 10^−9^
Charlson score	0.55	[0.3925: 0.6734]	5.13 × 10^−9^
ADL/6	−0.74	[−0.815: −0.6289]	6.38 × 10^−18^
Current weight in kilograms	−0.11	[−0.3041: 0.0879]	0.27
Height in cm	0.062	[−0.1379: 0.2575]	0.543
BMI	−0.14	[−0.3296: 0.0597]	0.168
Weight 6 months ago in kilograms	−0.051	[−0.2471: 0.1487]	0.616
Total number of medications	0.56	[0.4065: 0.6824]	2.08 × 10^−9^
Total number of therapeutic classes	0.6	[0.4516: 0.7108]	9.06 × 10^−11^
ZFS total	0.73	[0.6155: 0.8075]	3.01 × 10^−17^

ADL: activity daily living; BMI: body mass index; ZFS: Zulfiqar Frailty Scale.

- Age: The *p*-value (1.74 × 10^−9^) showed a significant positive correlation between age and SEGA scores, indicating that older individuals were more likely to be classified as frail according to the SEGA.
- Charlson score: The *p*-value (5.13 × 10^−9^) indicated a significant positive correlation between the Charlson comorbidity index and SEGA scores, suggesting that individuals with more comorbid conditions had higher SEGA scores.
- ADL/6: The *p*-value (6.38 × 10^−18^) showed a significant negative correlation between ADL scores and SEGA scores. Higher ADL scores were associated with lower SEGA scores, indicating that better functional ability is linked to lower frailty.
- Current weight in kilograms, height in cm, BMI, and weight 6 months ago in kilograms: The non-significant *p*-values indicated no significant correlation between these variables and SEGA scores.
- Total number of medications: The *p*-value (2.08 × 10^−9^) indicated a significant positive correlation, suggesting that a higher number of medications was associated with higher SEGA scores.
- Total number of therapeutic classes: The *p*-value (9.06 × 10^−11^) showed a significant positive correlation, linking polypharmacy to higher SEGA scores.
- ZFS total: The *p*-value (3.01 × 10^−17^) indicated a very strong positive correlation between the ZFS and SEGA scores, demonstrating that higher ZFS scores were significantly associated with higher SEGA scores.

### 3.2. Performance and Validity of the Zulfiqar Frailty Scale

Table 5 and Table 6 outline the outcomes of the comparison between ZFS and SEGA scores, predicated on the frailty status of the elderly subjects. The ZFS scale’s frailty detection threshold was established at three out of six criteria. In our investigation, our scale demonstrated a sensitivity of 64% and a negative predictive value of 80%. Notably, experiences of falls and hospitalizations within the past six months significantly influenced the classification of frailty according to both the ZFS and SEGA scales.

- Medical history of diabetes: The non-significant *p*-value (1) indicated no significant difference in frailty between those with and without a history of diabetes.
- Lives at home: The non-significant *p*-value (1) indicated no significant difference in frailty based on living at home status.
- Lives alone: The non-significant *p*-value (0.1079) indicated no significant difference in frailty based on living alone status.
- Presence of home care aides: The highly significant *p*-value (3.50 × 10^−14^) indicated that having home care aides was strongly associated with higher frailty scores.
- Weight loss > 5% in six months: The non-significant *p*-value (0.05572) suggested no significant difference in frailty based on recent weight loss.
- Does he/she have more than five therapeutic classes? The *p*-value (0.03428) showed a significant association between having more than five therapeutic classes and higher frailty scores.
- Successful monopodal support test (>5 s) on the right foot: The significant *p*-value (2.3913 × 10^−5^) indicated a strong association between failing the monopodal support test and higher frailty scores.
- Successful monopodal support test (>5 s) on the left foot: The significant *p*-value (1.8041 × 10^−6^) showed a strong association between failing the monopodal support test and higher frailty scores.
- Pathological monopodal support test: The significant *p*-value (6.12190 × 10^−6^) indicated a strong association between having a pathological monopodal support test and higher frailty scores.
- Does he/she complain of memory impairments? The significant *p*-value (1.2052 × 10^−8^) indicated a strong association between memory impairments and higher frailty scores.
- Has he/she been hospitalized in the last six months? The significant *p*-value (0.0054) suggested an association between recent hospitalization and higher frailty scores.
- Did he/she fall in the last six months? The non-significant *p*-value (0.0927) indicated no significant difference in frailty based on recent falls.
- mSEGA frail > 8: The highly significant *p*-value (1.95 × 10^−10^) indicated a strong association between being classified as frail by the SEGA and higher ZFS scores.

**Table 6:** Comparison of SEGA scores based on patient frailty.

SEGA	Frail, n = 28	Not Frail, n = 70	*p*-Value	Significant	Se	Sp	PPV	NPV	Youden Index
Medical history of diabetes			0.6211	n.s.					
Yes	9 (32%)	28 (40%)			32%	60%	24%	69%	−8%
No	19 (68%)	42 (60%)							
Lives at home			1	n.s.					
Yes	28 (100%)	70 (100%)			100%	0%	29%	/	/
No	0 (0%)	0 (0%)							
Lives alone			1	n.s.					
Yes	11 (39%)	26 (37%)			39%	63%	30%	72%	2%
No	17 (61%)	44 (63%)							
Presence of home care aides			2.1652 × 10^−8^	****					
Yes	21 (75%)	10 (14%)			75%	86%	68%	90%	61%
No	7 (25%)	60 (86%)							
Weight loss >5% in 6 months			0.0063	**					
Yes	6 (21%)	2 (2.9%)			21%	97%	75%	76%	18%
No	22 (79%)	68 (97%)							
Does he/she have more than 5 therapeutic classes?			0.0017	**					
Yes	13 (46%)	10 (14%)			46%	86%	57%	80%	32%
No	15 (54%)	60 (86%)							
Successful monopodal support test (>5 s) on the right foot?			0.002	***					
Yes	3 (11%)	36 (51%)			11%	49%	8%	58%	−40%
No	25 (89%)	34 (49%)							
Successful monopodal support test (>5 s) on the left foot?			2.5141 × 10^−5^	****					
Yes	2 (7%)	36 (51%)			7%	49%	5%	57%	−44%
No	26 (93%)	34 (49%)							
Pathological monopodal support test?			6.59650 × 10^−5^	****					
Yes	26 (93%)	36 (51%)			93%	49%	42%	94%	42%
No	2 (7%)	34 (49%)							
Does he/she complain of memory impairments?			0.0015	**					
Yes	21 (75%)	26 (37%)			75%	63%	45%	86%	38%
No	7 (25%)	44 (63%)							
Has he/she been hospitalized in the last 6 months?			0.0541	n.s.					
Yes	14 (50%)	19 (27%)			50%	73%	42%	78%	23%
No	14 (50%)	51 (73%)							
Did he/she fall in the last 6 months?			0.0011	**					
Yes	14 (50%)	11 (16%)			50%	84%	56%	81%	34%
No	14 (50%)	59 (84%)							

**** < 0.0001, *** < 0.001, ** < 0.01. Se: sensibility; Sp: specificity; PPV: positive predictive value; NPV: negative predictive value. n.s.: None significant.

- Medical history of diabetes: The non-significant *p*-value (0.6211) indicated no significant difference in frailty based on diabetes history.
- Lives at home: The non-significant *p*-value (1) indicated no significant difference in frailty based on living at home status.
- Lives alone: The non-significant *p*-value (1) indicated no significant difference in frailty based on living alone status.
- Presence of home care aides: The highly significant *p*-value (2.1652 × 10^−8^) indicated a strong association between having home care aides and higher frailty scores.
- Weight loss > 5% in six months: The significant *p*-value (0.0063) indicated an association between recent weight loss and higher frailty scores.
- Does he/she have more than five therapeutic classes? The significant *p*-value (0.0017) indicated an association between having more than five therapeutic classes and higher frailty scores.
- Successful monopodal support test (>5 s) on the right foot: The significant *p*-value (0.002) showed a strong association between failing the monopodal support test and higher frailty scores.
- Successful monopodal support test (>5 s) on the left foot: The significant *p*-value (2.5141 × 10^−5^) indicated a strong association between failing the monopodal support test and higher frailty scores.
- Pathological monopodal support test: The significant *p*-value (6.59650 × 10^−5^) indicated a strong association between having a pathological monopodal support test and higher frailty scores.
- Does he/she complain of memory impairments? The significant *p*-value (0.0015) indicated an association between memory impairments and higher frailty scores.
- Has he/she been hospitalized in the last six months? The non-significant *p*-value (0.0541) suggested no significant difference in frailty based on recent hospitalization.
- Did he/she fall in the last six months? The significant *p*-value (0.0011) indicated an association between recent falls and higher frailty scores.

**Figure 1:**
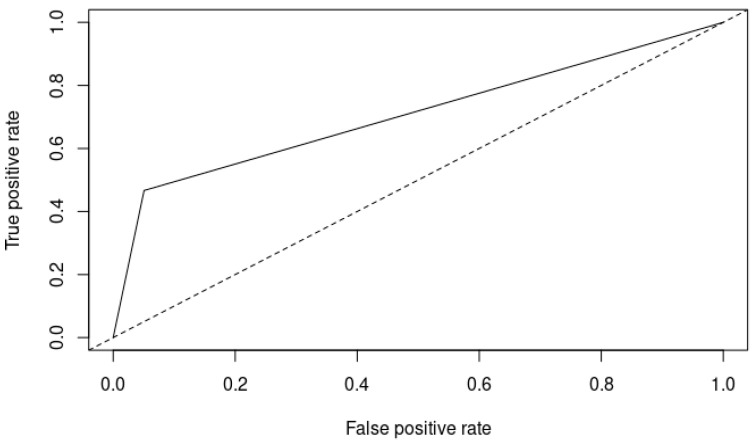
ROC curve of the Zulfiqar Frailty Scale (ZFS). Solid line: ROC curve of the classifier being evaluated: ZFS; Dotted line: Baseline representing a random classifier’s performance.

Lastly, the area under the curve (AUC) for the Zulfiqar Frailty Scale was reported as 0.8, as depicted in Figure 1.

## 4. Discussion

This study aimed to validate the Zulfiqar Frailty Scale (ZFS) and compare its effectiveness with the modified Short Emergency Geriatric Assessment (mSEGA) scale in identifying frailty among elderly patients in primary care settings. Our findings underscored the ZFS as a reliable and practical tool for frailty screening, showing significant correlations with the mSEGA scale and various geriatric parameters.

Key predictors of frailty identified in our study included age, comorbidity (Charlson score), polypharmacy (total number of medications and therapeutic classes), and functional ability (ADL scores).

Variables such as age, Charlson score, and polypharmacy were significantly correlated with both ZFS and SEGA scores, reinforcing their role as robust indicators of frailty. Non-significant correlations with weight, height, and BMI suggested that while these factors are important, they may not independently predict frailty in this cohort.

Significant associations with the presence of home care aides (*p* < 0.0001), monopodal support test results (*p* < 0.0001), memory impairments (*p* < 0.0001), and recent hospitalizations (*p* = 0.0054) underscored the multidimensional impact of frailty. These findings highlighted the importance of assessing not just physical health, but also social support and cognitive function in frailty screening.

Consistent with the ZFS, SEGA scores also showed significant associations with similar variables, validating the ZFS against a well-established tool. The high specificity and positive predictive value for home care aides and polypharmacy (*p* < 0.0001) emphasized these as critical areas for intervention.

Discussion on the Concordance and Correlation Between ZFS and Modified SEGA Scale

The strong positive correlation between ZFS and mSEGA (r = 0.73, *p* < 0.0001) supports the ZFS’s validity. This aligns with previous research emphasizing the need for comprehensive and multidimensional frailty assessment tools [3,7]. Both scales effectively captured the multifaceted nature of frailty, encompassing physical, cognitive, and social dimensions.

This value suggests that as scores on one scale increased, scores on the other scale tended to increase as well. Such a high correlation is indicative of substantial agreement between the two scales in identifying frailty, which aligns with findings in similar studies comparing different frailty assessment tools. For instance, studies comparing the Frailty Phenotype and Frailty Index have reported moderate to strong correlations, underscoring the reliability of these tools in clinical settings [13].

Influence of Falls and Hospitalizations

The significant impact of falls and hospitalizations on frailty classification according to both ZFS and mSEGA scales is a noteworthy finding. These factors are well-documented predictors of frailty and adverse outcomes in older adults. Research has consistently shown that a history of falls and hospitalizations are strongly associated with increased frailty and higher risks of subsequent morbidity and mortality [14,15,16].

Area Under the Curve (AUC)

The AUC of 0.8 for the ZFS suggested excellent discriminative ability in distinguishing between frail and non-frail individuals. An AUC of 0.8 is generally considered good and implies that the ZFS has a high level of accuracy in predicting frailty. This performance is comparable to other validated frailty scales. For example, the Clinical Frailty Scale (CFS) and the Frailty Index (FI) often report AUC values in the range of 0.75 to 0.85, demonstrating their effectiveness in clinical practice [7,8,9,10,11,12,13,14,15,16,17].

The findings of this study contribute to the growing body of evidence supporting the use of multiple frailty assessment tools in clinical settings. The strong correlation between ZFS and mSEGA, coupled with the good AUC, suggests that the ZFS is a reliable tool for frailty screening. However, its moderate sensitivity highlights the need for further refinement to enhance its diagnostic accuracy. The significant influence of falls and hospitalizations reinforces the importance of incorporating these factors into frailty assessments to improve the prediction of adverse outcomes.

Overall, this study’s innovative approach of comparing ZFS with mSEGA offers valuable insights into the effectiveness of different frailty assessment tools, paving the way for more personalized and accurate interventions in geriatric care.

Comparative Tools and Broader Context

Comparative tools, such as the Fried Frailty Phenotype and the Rockwood Frailty Index, have been extensively used in various settings. However, their application in primary care is limited by practical constraints, such as the need for equipment and longer administration times [4,6,7]. The ZFS addresses these limitations, providing a viable alternative for busy clinical environments.

Recent studies highlight the increasing importance of frailty screening in primary care to prevent adverse outcomes, such as hospitalization, falls, and mortality [18,19]. Integrating the ZFS into routine practice can help identify at-risk individuals early, allowing for timely interventions.

A subsequent study comparing the Zulfiqar Frailty Scale to the SEGA scale confirmed the initial results [20]. With a sensitivity of 64%, a negative predictive value of 80%, and an AUC of 0.8, the Zulfiqar Frailty Scale exhibited both good performance and relevance, correlating well with the SEGA scale. Regarding the goal of adapting to ambulatory care, this frailty scale seems well suited. It does not require prior training for healthcare providers or additional equipment, such as a dynamometer for the Fried scale, which can be a financial hurdle. The average administration time was under 2 min (around 90 s in this study). In contrast, the modified SEGA score can take 4 to 10 min [20,21,22], which was also observed in our study. Considering that the average consultation time in general medicine is 15–16 min or longer for poly-pathological elderly subjects [23], this time discrepancy matters. It could thus be a significant advantage for large-scale screening, especially during quarterly prescription renewal visits or home visits.

Limitations and Future Research

While this study provided valuable insights, its observational design and relatively small sample size limit the generalizability of the findings. Future research should focus on larger, diverse populations to validate these results further. Longitudinal studies are also needed to assess the ZFS’s predictive validity for long-term health outcomes.

Moreover, exploring the integration of the ZFS with electronic health records (EHR) could enhance its utility, enabling automated frailty screening and monitoring in primary care. Further research should also investigate the ZFS’s applicability in different healthcare settings, including specialized geriatric care and community-based programs.

## 5. Conclusions

Ongoing research is focused on assessing the ZFS’s reproducibility and performance in predicting adverse outcomes under stress. We eagerly anticipate sharing the results of these investigations in the upcoming weeks.

## Figures and Tables

**Table 1:** Criteria of the Zulfiqar Frailty Scale [1].

Is there a weight loss ≥ 5% in 6 months?	Yes	No
Monopodal support test < 5 s?	Yes	No
Does he/she live alone at home?	Yes	No
Limitation in activities of daily living: requires caregivers at home?	Yes	No
Does he/she complain of memory impairment? (answer can be given by a caregiver)	Yes	No
Has he/she been issued a prescription with more than 5 therapeutic classes (≥5) within the last 6 months?	Yes	No

**Table 2:** Population characteristics.

Population Characteristics (n = 98)		
	Proportions	Mean (Standard
	(%)	Deviation)
Age	/	75 (8)
Sex		
Female	67 (68%)	/
Male	31 (32%)	/
Medical history of diabetes		
Yes	37 (38%)	/
No	61 (62%)	/
Charlson score	/	4.06 (1.62)
Marital status, single		
Yes	16 (16%)	/
No	82 (84%)	/
Marital status, widowed		
Yes	28 (29%)	/
No	70 (71%)	/
Living at home		
Yes	98 (100%)	/
No	0 (0%)	/
Lives alone		
Yes	37 (38%)	/
No	61 (62%)	/
ADL/6	/	5.61 (0.62)
Presence of home care assistance		
Yes	31 (32%)	/
No	67 (68%)	/
Current weight in kilograms	/	75 (17)
Height in cm	/	165 (9)
BMI	/	28 (7)
Weight 6 months ago in kilograms	/	76 (17)
Weight loss > 5% in 6 months		
Yes	8 (8.2%)	/
No	90 (92%)	/
Total number of medications	/	5.02 (2.87)
Total number of therapeutic classes	/	3.30 (1.92)
Does he/she have more than 5 therapeutic classes?		
Yes	23 (23%)	/
No	75 (77%)	/
Successful monopodal support test (>5 s) on right foot?		
Yes	39 (40%)	/
No	59 (60%)	/
Successful monopodal support test (>5 s) on left foot?		
Yes	38 (39%)	/
No	60 (61%)	/
Pathological monopodal support test?		
Yes	62 (63%)	/
No	36 (37%)	/
Does he/she complain of memory impairments?		
Yes	47 (48%)	/
No	51 (52%)	/
Has he/she been hospitalized in the last 6 months?		
Yes	33 (34%)	/
No	65 (66%)	/
Has he/she had any falls in the last 6 months?		
Yes	25 (26%)	/
No	73 (74%)	/
mSEGA grid A (/26)	/	6.2 (3.8)
SEGA frail > 8		
Yes	28 (29%)	/
No	70 (71%)	/
ZFS total	/	2.16 (1.39)
ZFS frail ≥ 3/6		
Yes	39 (40%)	/
No	59 (60%)	/

ADL: activity daily living; mSEGA: modified Short Emergency Geriatric Assessment; ZFS: Zulfiqar Frailty Scale; BMI: body mass index.

**Table 3:** Pearson correlation coefficients with ZFS score variable.

Variables	Pearson Correlation Coefficient	CI	*p*-Value
Age	0.41	[0.2355: 0.5664]	0.000022
Charlson score	0.4	[0.2214: 0.5562]	0.0000405
ADL/6	−0.52	[−0.6517: −0.3593]	3.91 × 10^−8^
Current weight in kilograms	−0.08	[−0.2751: 0.1192]	0.427
Weight in cm	−0.032	[−0.2287: 0.1677]	0.756
BMI	−0.08	[−0.272: 0.1226]	0.447
Weight 6 months ago in kilograms	−0.03	[−0.2273: 0.1692]	0.768
Total number of medications	0.54	[0.3853: 0.6687]	8.09 × 10^−9^
Total number of therapeutic classes	0.52	[0.3626: 0.6539]	3.21 × 10^−8^
mSEGA scale grid A (/26)	0.73	[0.6155: 0.8075]	3.01 × 10^−17^

ADL: activity daily living; BMI: body mass index; mSEGA: modified Short Emergency Geriatric Assessment.

**Table 5:** Comparison of ZFS scores based on patient frailty.

Zulfiqar Frailty Scale (ZFS)	Frail, n = 39	Not Frail, n = 59	*p*-Value	Significant	Se	Sp	PPV	NPV	Youden Index
Medical history of diabetes			1	n.s.					
Yes	15 (38%)	22 (37%)			38%	63%	41%	61%	1%
No	24 (62%)	37 (63%)							
Lives at home			1	n.s.					
Yes	39 (100%)	59 (100%)			100%	0%	40%	/	/
No	0 (0%)	0 (0%)							
Lives alone			0.1079	n.s.					
Yes	19 (49%)	18 (31%)			49%	69%	51%	67%	18%
No	20 (51%)	41 (69%)							
Presence of home care aides			3.50 × 10^−14^	**					
Yes	29 (74%)	2 (3%)			74%	97%	94%	85%	71%
No	10 (26%)	57 (97%)							
Weight loss > 5% in 6 months			0.05572	n.s.					
Yes	6 (15%)	2 (3%)			15%	97%	75%	63%	12%
No	33 (85%)	57 (97%)							
Does he/she have more than 5 therapeutic classes?			0.03428	*					
Yes	14 (36%)	9 (15%)			36%	85%	61%	67%	21%
No	25 (64%)	50 (85%)							
Successful monopodal support test (>5 s) on the right foot?			2.3913 × 10^−5^						
Yes	5 (13%)	34 (58%)			13%	42%	13%	42%	−45%
No	34 (87%)	25 (42%)							
Successful monopodal support test (>5 s) on the left foot?			1.8041 × 10^−6^	****					
Yes	4 (10%)	34 (58%)			10%	42%	11%	42%	−48%
No	35 (90%)	25 (42%)							
Pathological monopodal support test?			6.12190 × 10^−6^	****					
Yes	35 (90%)	27 (46%)			90%	54%	56%	89%	44%
No	4 (10%)	32 (54%)							
Does he/she complain of memory impairments?			1.2052 × 10^−8^	****					
Yes	33 (85%)	14 (24%)			85%	76%	70%	88%	61%
No	6 (15%)	45 (76%)							
Has he/she been hospitalized in the last 6 months?			0.0054	**					
Yes	20 (51%)	13 (22%)			51%	78%	61%	71%	29%
No	19 (49%)	46 (78%)							
Did he/she fall in the last 6 months?			0.0927	n.s.					
Yes	14 (36%)	11 (19%)			36%	81%	56%	66%	17%
No	25 (64%)	48 (81%)							
mSEGA frail > 8			1.95 × 10^−10^	****					
Yes	25 (64%)	3 (5%)			64%	95%	89%	80%	59%
No	14 (36%)	56 (95%)							

**** < 0.0001, ** < 0.01, * < 0.05. Se: sensibility; Sp: specificity; PPV: positive predictive value; NPV: negative predictive value. n.s.: None significant.

## Data Availability

The datasets used and/or analyzed during the current study are available from the corresponding author upon request.
